# Eruptive Collagenoma: A Rare Encounter in Clinical Dermatology

**DOI:** 10.7759/cureus.100338

**Published:** 2025-12-29

**Authors:** Fatemah F Bousheheri, Alsadat Mosbeh, Abeer Albazali, Mariam Alshammari

**Affiliations:** 1 Dermatology, Al-Amiri Hospital, Kuwait City, KWT; 2 Dermatology/Dermatopathology, Al-Azhar University, Cairo, EGY; 3 Dermatology, Farwaniya Hospital, Sabah Al Nasser, KWT; 4 Dermatology, Dermatology Clinic, Kuwait City, KWT

**Keywords:** connective tissue, connective tissue nevus, eruptive collagenoma, rare skin disease, skin papule

## Abstract

Eruptive collagenoma is a rare variant of connective tissue nevus characterized by the sudden appearance of multiple, asymptomatic, skin-colored papules, nodules, or plaques. These lesions typically occur on the trunk, upper extremities, or head and neck, most often during adolescence or early adulthood. The exact etiology and incidence remain unclear, and no consistent familial or systemic associations have been identified. We report a rare case of eruptive collagenoma presenting with multiple elevated, skin-colored papular and nodular lesions on the upper back of a young adult woman.

## Introduction

Eruptive collagenoma is an uncommon acquired connective tissue nevus characterized by excessive collagen deposition within the dermis and a relative reduction of elastic fibers. Clinically, it presents as multiple, firm, skin-colored papules or nodules that develop over time, most often on the trunk or upper extremities, without a family history or systemic involvement [1,2]. The exact pathogenesis remains uncertain; altered fibroblast activity and reduced collagenase function have been proposed mechanisms [3].

Reported cases most frequently present in adolescence and early adulthood, and the distribution is usually on the trunk (particularly the upper back) and proximal limbs [4]. Although the condition is benign, it is easily confused with other eruptive papular disorders and with elastic-tissue nevi. Clear clinicopathologic correlation is therefore important, and additional well-documented cases help refine recognition of this rare entity [4].

## Case presentation

A 28-year-old woman presented with a three-year history of gradual onset and slow progression of multiple, asymptomatic, skin-colored papules and nodules on the upper back. There was no history of preceding trauma, prior cutaneous lesions, or similar complaints among family members. She denied systemic symptoms and had no known history of malignancy or chronic systemic disease. General and systemic examinations were unremarkable.

**Figure 1:**
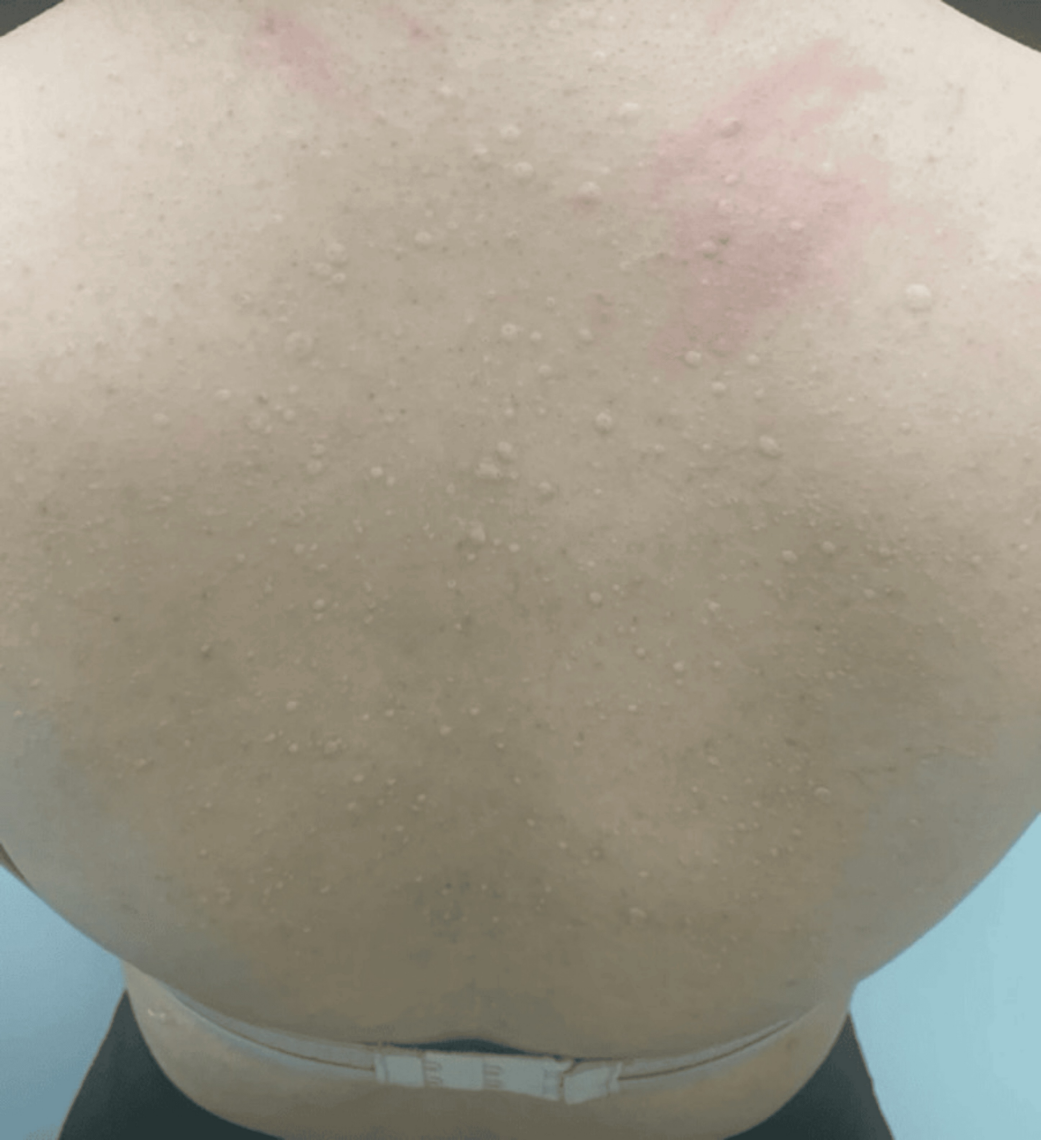
Multiple discrete, firm, non-tender, skin-colored papules and nodules symmetrically distributed over the upper back.

Dermatological examination revealed multiple, discrete, firm, non-tender papules and nodules, skin-colored, symmetrically distributed over the upper back (Figure 1).

Based on morphology and distribution, the clinical differential diagnosis included papular elastorrhexis, elastic nevus, nevus anelasticus, keloids, and anetoderma. Several features made these less likely: lesions lacked the soft, herniating quality typical of anetoderma; there was no antecedent injury or expanding plaque to suggest keloids; and the papules were uniformly firm and skin-colored rather than variably atrophic or wrinkled. Additional causes of eruptive firm nodules (such as dermatofibromas, neurofibromas, and leiomyomas) were considered but were not supported by the clinical appearance (absence of pigmentation or dimple sign, no buttonhole sign, and no pain).

**Figure 2:**
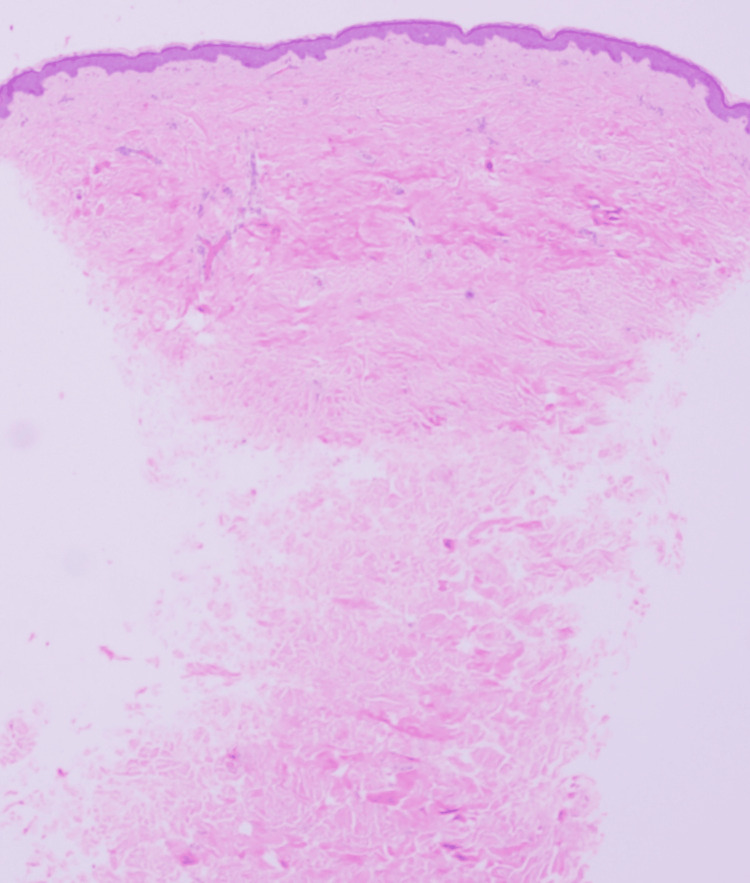
Histopathological examination showing a thinned epidermis and dense, interlacing bundles of fibrocollagenous tissue within the dermis on hematoxylin and eosin staining.

**Figure 3:**
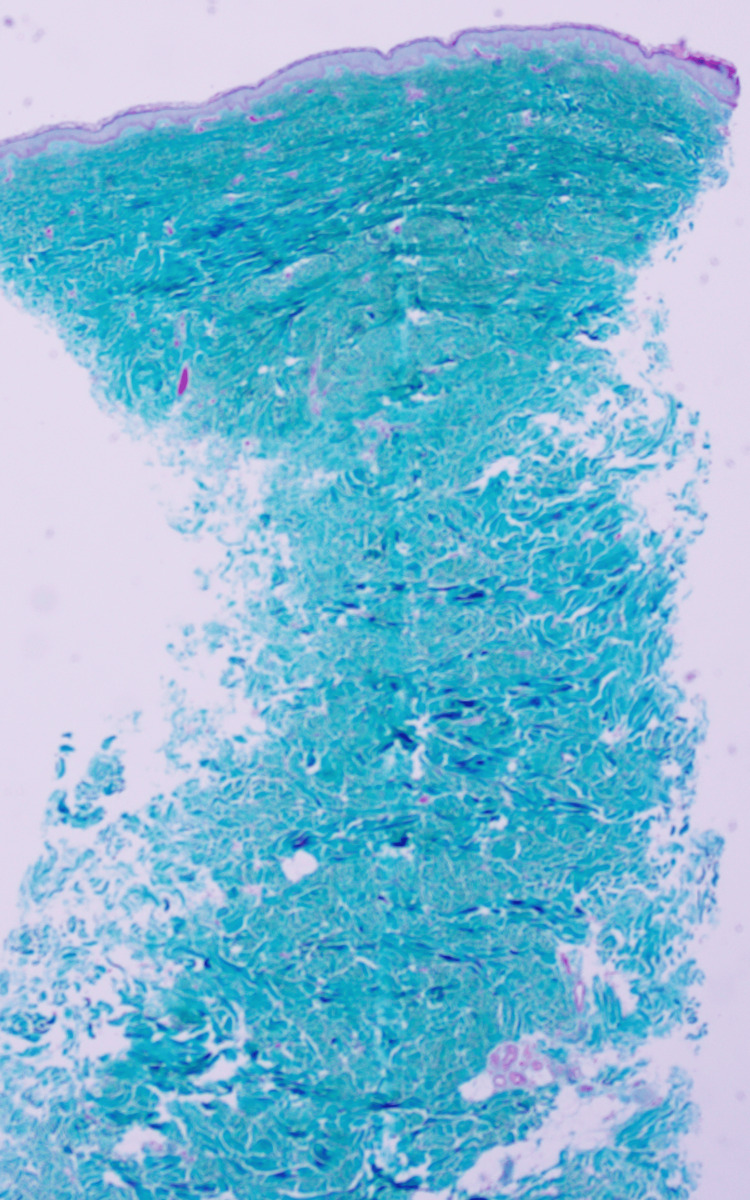
Masson’s trichrome special staining (blue) demonstrating abundant collagen fibers in the dermis, indicating increased collagen deposition.

**Figure 4:**
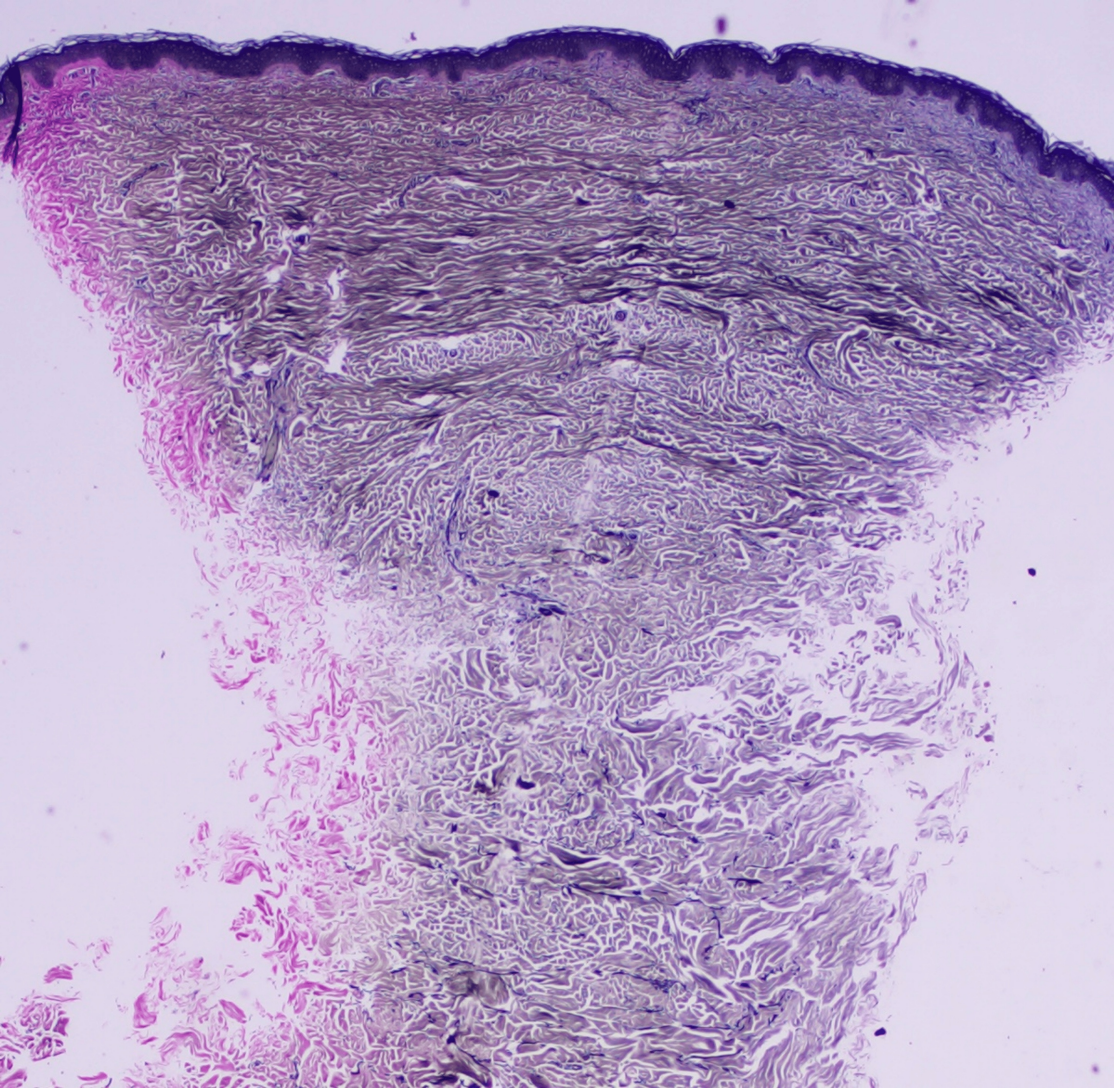
Van Gieson special staining (red) demonstrating abundant collagen fibers in the dermis with markedly reduced and fragmented elastic fibers.

Laboratory testing was within normal limits (Table 1). A punch biopsy was performed. Hematoxylin and eosin sections showed a thinned epidermis overlying a dermis expanded by dense, interlacing bundles of fibrocollagenous tissue (Figure 2). Special stains highlighted the collagen-rich nature of the lesion: Masson's trichrome demonstrated increased dermal collagen (Figure 3), and Van Gieson staining showed abundant collagen with markedly reduced and fragmented elastic fibers (Figure 4), supporting the diagnosis of eruptive collagenoma.

**Table 1:** Patient laboratory test results. CRP: C-reactive protein; ESR: erythrocyte sedimentation rate; AST: aspartate aminotransferase; ALT: alanine aminotransferase

Test	Patient result	Reference range
CRP	8	<10 mg/L
ESR	10	<15 mm/hr
AST	31	10-40 U/L
ALT	45	7-56 U/L

Special stains with Masson’s trichrome (blue) and Van Gieson (red) demonstrated abundant collagen fibers in the dermis (Figures 3, 4). Elastic fibers appeared markedly reduced and fragmented, confirming the diagnosis of eruptive collagenoma.

## Discussion

Collagenomas are benign connective tissue nevi composed predominantly of collagen. They are commonly classified by mode of inheritance (acquired versus inherited) and by distribution (localized versus generalized) [1]. The acquired, eruptive form typically presents as multiple asymptomatic, flesh-colored papules or nodules on the trunk or proximal extremities, usually without systemic disease or family history [4]. In contrast, inherited forms include familial cutaneous collagenoma and collagenomas seen in tuberous sclerosis complex (e.g., shagreen patches), which may present in the second or third decades and can be accompanied by syndromic/systemic features [2].

Age and anatomic distribution

Age of onset and anatomic distribution can be clinically useful. Most reported eruptive cases occur in the first two decades of life, but adult-onset presentations have been described [4]. The trunk, especially the upper back, is a common site; less typical locations (palms, soles, genital region, or unilateral distribution) have also been reported [3].

Clinical differential diagnosis

Eruptive collagenoma should be differentiated from other causes of multiple firm papules or nodules, particularly elastic-tissue disorders and benign fibrous proliferations [4]. Helpful bedside distinctions include papular elastorrhexis/nevus anelasticus (often smaller papules; diagnosis is driven by elastic-fiber changes on histology), keloids (history of trauma and plaques extending beyond the original injury), anetoderma (soft, herniating lesions), dermatofibroma (often pigmented with a dimple sign), neurofibroma (softer lesions with a buttonhole sign), and cutaneous leiomyoma (characteristically painful).

Syndromic and systemic associations

When collagenomas are numerous, early-onset, familial, or accompanied by other characteristic skin findings, syndromic associations should be considered. Collagenomas/connective tissue nevi are described in tuberous sclerosis complex (where a shagreen patch represents a connective tissue nevus) [2], Buschke-Ollendorff syndrome (connective tissue nevi with osteopoikilosis) [5], and in some patients with multiple endocrine neoplasia type 1 (MEN1) who have multiple collagenomas along with other cutaneous lesions [5]. Sporadic associations with systemic conditions (including cardiomyopathy, Down syndrome, hypogonadism, and pseudohypoparathyroidism) have also been reported [5]. In our patient, there was no supportive family history, systemic symptomatology, or examination findings, favoring an isolated eruptive collagenoma.

Histopathology: key differentials

Histopathologic diagnosis can be challenging because several benign fibrous dermal lesions demonstrate increased collagen. Collagenoma typically shows thickened, haphazardly arranged collagen bundles with a relative reduction or fragmentation of elastic fibers on elastic/collagen stains [1,4], as seen in this case. Sclerotic fibroma (storiform collagenoma) is generally well circumscribed and hypocellular, with thick collagen bundles arranged in a whorled "plywood-like" pattern and prominent clefting, whereas sclerotic variants of dermatofibroma/fibroma more often show a spindle-cell proliferation with peripheral collagen trapping, sometimes with associated epidermal changes. Accordingly, clinicopathologic correlation and explicit consideration of these entities strengthen diagnostic confidence.

Atypical anatomic presentations

In addition to the typical truncal distribution, eruptive collagenomahas have been reported in unusual locations, including the face [6] and the lower extremities, such as the calf [7]. These reports underscore variability in anatomic presentation and support careful clinical and histopathologic evaluation when distribution is atypical.

Management

There is no standardized treatment for eruptive collagenoma because lesions are benign and usually asymptomatic [4,8]. Management is therefore conservative, focusing on reassurance and observation [4,8]. For patients seeking cosmetic improvement, reported options include procedural removal by punch or excisional surgery; dermabrasion and laser therapy have also been described [8]. For larger or more prominent nodules, complete surgical excision is the most definitive approach when feasible, with counseling regarding scar risk based on anatomic site [8]. In our patient, no active intervention was initiated; the patient was counseled about the benign course and advised to return for follow-up if new lesions developed or existing lesions changed.

## Conclusions

Eruptive collagenoma is a rare benign connective tissue nevus that may mimic several eruptive papular disorders. Recognition relies on clinicopathologic correlation, including demonstration of increased dermal collagen with reduced elastic fibers. When lesions are extensive or accompanied by suggestive findings, syndromic associations should be considered. Management is usually conservative, with excision reserved for select lesions causing symptoms or cosmetic concern.
